# Systematic Elucidation of the Mechanism of Genistein against Pulmonary Hypertension via Network Pharmacology Approach

**DOI:** 10.3390/ijms20225569

**Published:** 2019-11-07

**Authors:** Yucai Chen, Di Chen, Sijia Liu, Tianyi Yuan, Jian Guo, Lianhua Fang, Guanhua Du

**Affiliations:** 1 School of Traditional Chinese Medicine, Beijing University of Chinese Medicine, Beijing 100029, China; 201801009@bucm.edu.cn (Y.C.); 20170931107@bucm.edu.cn (S.L.); 2 State Key Laboratory of Bioactive Substance and Function of Natural Medicines, Institute of Materia Medica, Chinese Academy of Medical Sciences and Peking Union Medical College, Beijing 100050, China; chendi@imm.ac.cn (D.C.); yuantianyi@imm.ac.cn (T.Y.)

**Keywords:** genistein, pulmonary hypertension, network pharmacology, peroxisome proliferator-activated receptor γ (PPARγ), molecular docking

## Abstract

Numerous studies have shown that genistein has a good therapeutic effect on pulmonary hypertension (PH). However, there has been no systematic research performed yet to elucidate its exact mechanism of action in relation to PH. In this study, a systemic pharmacology approach was employed to analyze the anti-PH effect of genistein. Firstly, the preliminary predicted targets of genistein against PH were obtained through database mining, and then the correlation of these targets with PH was analyzed. After that, the protein-protein interaction network was constructed, and the functional annotation and cluster analysis were performed to obtain the core targets and key pathways involved in exerting the anti-PH effect of genistein. Finally, the mechanism was further analyzed via molecular docking of genistein with peroxisome proliferator-activated receptor γ (PPARγ). The results showed that the anti-PH effect of genistein may be closely related to PPARγ, apoptotic signaling pathway, and the nitric oxide synthesis process. This study not only provides new insights into the mechanism of genistein against PH, but also provides novel ideas for network approaches for PH-related research.

## 1. Introduction

As a serious cardiopulmonary disease with increased morbidity and mortality, pulmonary hypertension (PH) is characterized by pulmonary vascular remodeling and right heart failure due to increased afterload. Clinically, PH can be divided into five different types according to the cause of the disease [1]. The major pathology associated with PH is pulmonary circulation disturbance caused by pulmonary vascular remodeling, which leads to an increase in pulmonary blood pressure. At present, there are three main types of drugs for treating PH in the clinic, including phosphodiesterase 5 (PDE-5) inhibitors (sildenafil, tadalafil), prostacyclin analogues (epoprostanol, treprostinil), and endothelin receptor antagonists (bosentan, ambrisentan). The diagnosis and treatment of PH has made significant progress in recent years, and existing targeted drugs have significantly improved the survival rate and quality of life for PH patients. However, PH is still an intractable disease to cure, and it is an urgent matter to find new therapeutic targets, so as to develop new therapeutic drugs [2,3].

Complex diseases such as PH are not caused by alterations in a single gene, but by an accumulation of multiple genetic defects and functional variants [4]. Network pharmacology is a discipline for investigating pathogenesis of diseases through constructing and analyzing biological networks [5]. The networks shed light on the interaction between drugs and their targets, and provide direction for the discovery of new therapeutic agents. This method is especially well suited for studying complex diseases such as PH [6,7].

Genistein is an isoflavone compound, which is extracted from legumes such as soybean, puerarin, and sassafras. It exhibits an estrogen-like effect such as with daidzein and puerarin. A large body of evidences suggest that genistein has a wide range of pharmacological effects on the cardiovascular system. Long-term intake of genistein can improve the ejection function of the left ventricle in postmenopausal women with metabolic syndrome [8]. In the aortic coarctation-induced myocardial fibrosis model, genistein can improve cardiac function and reduce collagen deposition by regulating the metastasis-associated gene 3 / TGFβ-activated kinase 1 (MTA3/TAK1) signaling pathway [9]. Genistein has been found to inhibit the development of PH in a variety of models in vitro and in vivo. In the model of monocrotaline (MCT)-induced PH, genistein can increase animal survival rate in a dose-dependent manner and can postpone vascular remodeling [10]. Genistein can significantly inhibit hypoxia-induced hypertrophy of pulmonary artery smooth muscle cells in vitro [11]. In the low temperature-induced broiler PH model, genistein can also delay disease progression by improving endothelial cell function [12].

In light of the above-mentioned studies, it can be seen that genistein plays a significant preventive and therapeutic role in PH induced through different targets [10,11,12]. Mechanistically, the effects of genistein are related to various targets and signaling pathways. In order to explain the role of genistein against PH comprehensively, this study employed the method of network pharmacology to investigate the targets closely related to genistein in treating PH. 

## 2. Results

### 2.1. Druglikeness Analysis of Genistein

Lipinski’s rule of five (RO5) describes molecular properties, which are important for a drug's pharmacokinetics in the human body, including their absorption, distribution, metabolism, and excretion. According to the RO5, a drug-like compound should have a molecular weight of less than 500 g/mol, a polar surface area (PSA) of less than or equal to 140 ^A, a computed octanol/water partition coefficient (XLogP3-AA) of less than 5, less than 10 rotatable bonds (RB), no more than 10 hydrogen bond acceptor (HBA), and no more than 5 hydrogen bond donors (HBD) [13]. According to the parameters from PubChem database, the properties of genistein were in line with RO5, indicating that it had good drug-like properties. 

The ADMET descriptor gives four prediction levels within the 95% and 99% confidence ellipsoids for HIA and BBB. The four levels for HIA are 0 (good), 1 (moderate), 2 (poor), 3 (very poor); whereas for BBB, 0 (very high penetrant), 1 (high), 2 (medium), 3 (low) and 4 (undefined). As shown in Table 1, genistein was well absorbed through the intestine and had low BBB penetration.

### 2.2. Correlation Analysis between Targets of Genistein and PH

A total of 22 intersection genes of genistein and PH were input into the VarElect online tool for analysis of genotype-phenotype correlations. The result is shown in Table 2. Nitric oxide synthase (NOS) is a family of enzymes that catalyzes the production of nitric oxide (NO) from L-arginine. There are three subtypes of NOS in mammals: NOS1 (neuronal NOS, nNOS), NOS2 (inducible NOS, iNOS), and NOS3 (endothelial NOS, eNOS). These series of subtypes are involved in a wide range of physiological functions in the cardiovascular, immune, and nervous systems [14]. Vascular endothelial growth factor (VEGF), also known as vascular permeability factor, is a highly specific factor that promotes the growth of vascular endothelial cells and increases vascular permeability, extracellular matrix degeneration, vascular endothelial cell migration, proliferation, and angiogenesis [15]. Prostaglandin-endoperoxide synthase (PTGS), also known as cyclooxygenase, is a key enzyme in the initial steps of prostaglandin synthesis [16]. Peroxisome proliferator-activated receptor γ (PPARγ) is a type II nuclear receptor encoded by the PPARG gene in humans. PPARγ has been implicated in the pathology of numerous diseases including obesity, diabetes, hypertension, and cancer; it also plays a key role in the pathological process of PH [17,18].

### 2.3. Protein–Protein Interaction Network

**Figure 1:**
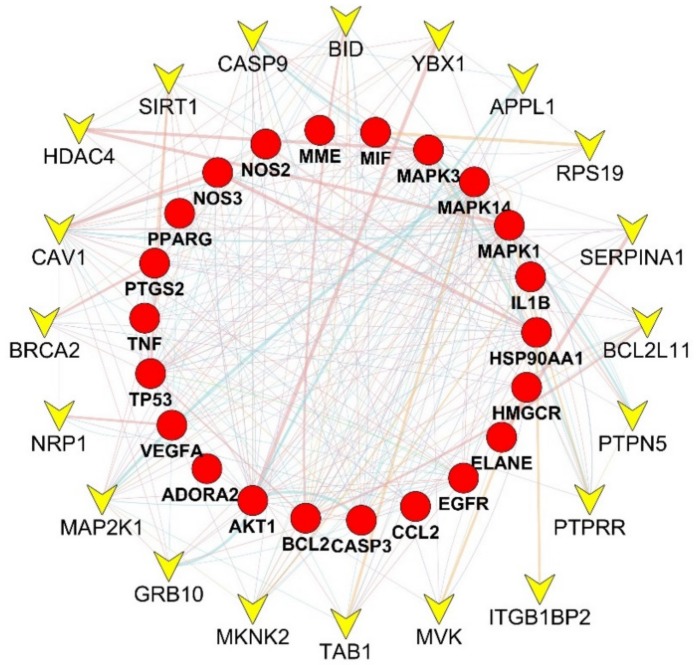
Network of potential targets of genistein against PH analyzed by GeneMANIA. Genes on the inner ring were submitted as query terms in searches. Nodes on the outer ring indicate genes associated with query genes. Functional association of targets was analyzed, and different color of connecting lines represent different correlations.

In order to further supplement other related genes in the network and find the key signaling pathways, we imported 22 target genes into the GeneMANIA tool to obtain a PPI network. GeneMANIA uses a database of organism-specific weighted networks to construct the resulting composite network. The source networks are grouped by type (e.g., co-expression) and list each network weight, as well as the sum of the weights of the networks in each group. The percentage in the results stands for the weight of interaction relationship in the network. Our result revealed that in the interaction between targets of the network, 34.98% were co-expression and 26.33% had physical interactions. There were also relationships of co-localization and shared protein domains (Figure 1). The calculated average shortest path length, betweenness centrality, closeness centrality, and degree of nodes in the network are shown in Table S3. Through the analysis of network topology parameters, it is found that the ranking of MAPK family genes (MAPK1, MAPK3 and MAPK14), NO pathway related genes (AKT1, NOS2, NOS3 and PPARG), ADORA2A, and HSP90AA1 was highly important in the network.

### 2.4. GO and Pathway Analysis

**Figure 2:**
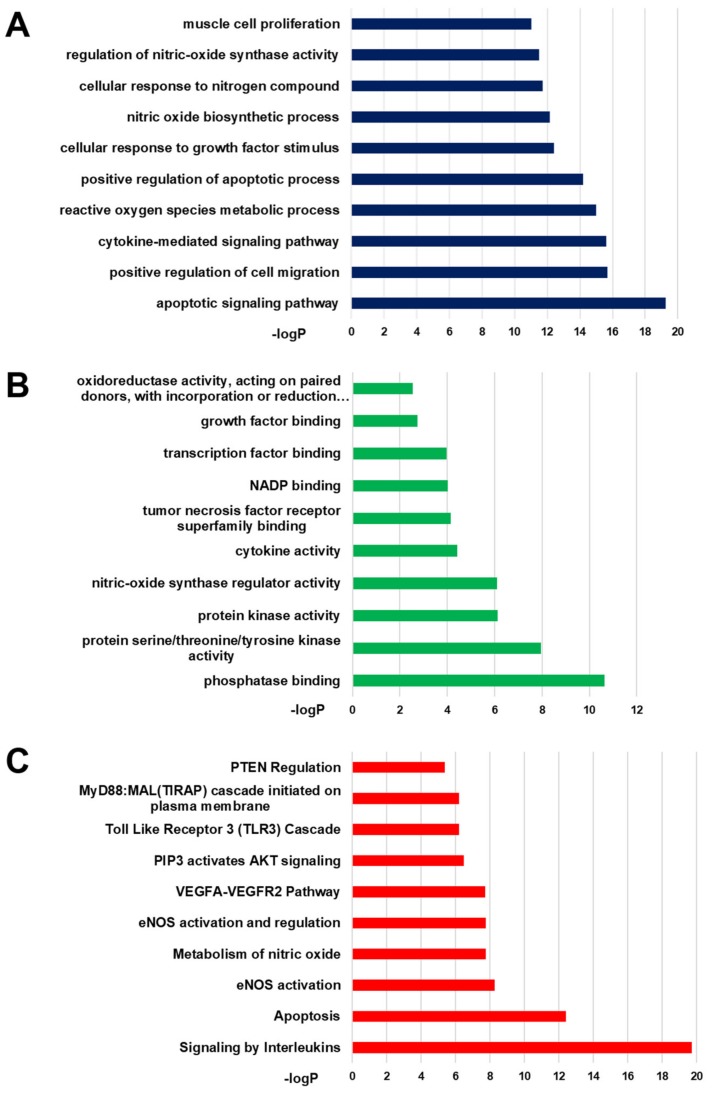
Gene Ontology (GO) analysis and Reactome pathway analysis of genistein targets. The y-axis shows significantly enriched (**A**) “Biological Process” (BP) categories, (**B**) “Molecular function” (MF) categories and (**C**) “Reactome Gene Sets” associated with the targets; the x-axis shows the enrichment scores of these terms.

To further analyze the function of genistein targets, we carried out GO analysis for a total of 42 targets. In categories related to the pathogenesis of PH, these targets not only modulated cell migration, apoptotic process, and muscle cell proliferation (Figure 2A), but also involved in nitric-oxide synthase, growth factor binding, and oxidoreductase activity (Figure 2B). Reactome pathway analysis was used to explore the potential pathways affected by genistein. Results demonstrated that “signaling by interleukins”, “metabolism of nitric oxide”, “PIP3 activates Akt signaling”, and “Toll like receptor 3 (TLR3) cascade” were obviously enriched. The above molecular functions and biological processes were closely related to the occurrence and development of pulmonary hypertension. Therefore, these data provided theoretical evidences that the effect of genistein against PH was possibly linked with the nitric oxide production process and closely related to smooth muscle proliferation process and inflammatory response.

### 2.5. Target-Function Network (T-F Network)

**Figure 3:**
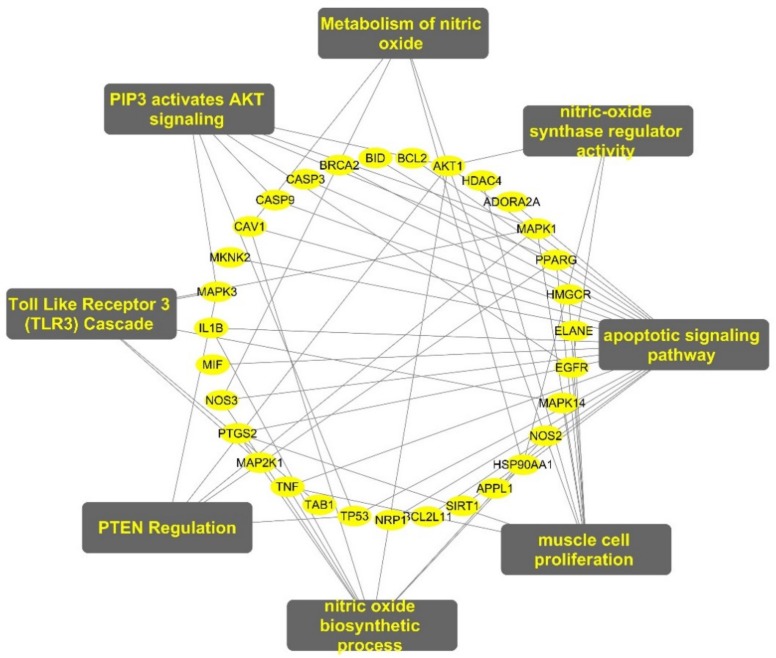
Target-function network. A functional module is linked to the targets if the target is involved in that biological process or pathway.

We conducted further network analysis on several representative signal pathways, biological processes and molecular function, and constructed the target-pathway/function network. As shown in Figure 3, many targets were involved in multiple biological processes simultaneously. For example, PPARG was involved in both “muscle cell proliferation”, “PIP3 activates AKT signaling”, and “PTEN Regulation” processes. NOS3 belonged to both “nitric oxide biosynthetic process” and “apoptotic signaling pathway”. AKT1 was involved in many biological processes such as “muscle cell proliferation”, “PIP3 activates AKT signaling”, and “metabolism of nitric oxide”. The above results indicate that genistein can exert an anti-PH effect through multiple targets and pathways, so genistein may be suitable for the complex mechanism of pulmonary hypertension.

### 2.6. Molecular Docking

**Figure 4:**
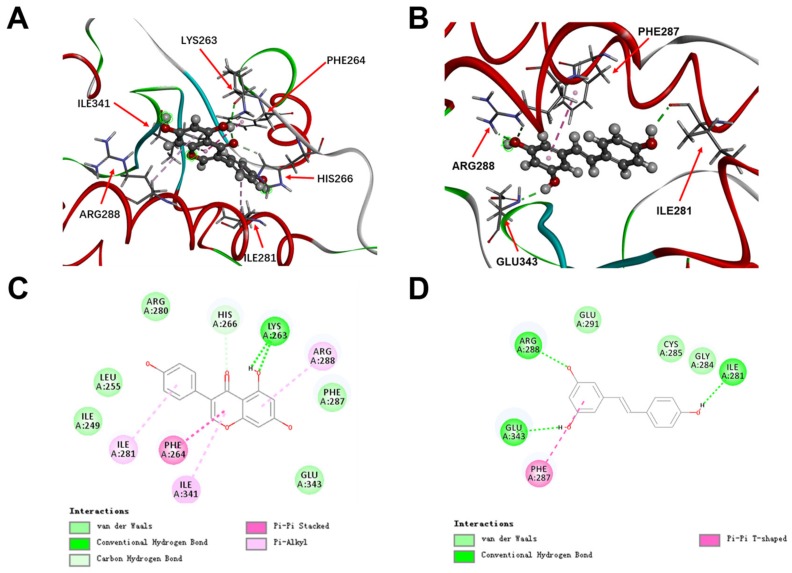
The docking patterns of genistein and trans-resveratrol interact with PPARγ in the active site of PPARγ illustrated by CDOCKER. 3D presentation of interaction between genistein (**A**) or trans-resveratrol (**B**) and PPARγ. 2D presentation of interaction between genistein (**C**) or trans-resveratrol (**D**) and PPARγ. The red arrows refer to the amino acid residues that interact with genistein and tran-resveratrol in the binding site of PPARγ.

To test the reliability of the key drug-target interactions and explore the accurate binding modes, we selected PPARγ as the target for molecular docking based on above results of network pharmacology and existing experimental studies about genistein against PH. The result shows that there was stronger interaction between genistein and PPARγ with the CDOCKER energy of 29.4126 kcal/mol, while the CDOCKER energy between trans-resveratrol and PPARγ was 25.1709 kcal/mol (Table 3). The table shows two parameters: CDOCKER energy and CDOCKER interaction energy; both are used to sort the poses of each input ligand. But CDOCKER energy is more comprehensive because it includes internal ligand strain energy besides CDOCKER interaction energy. In terms of interaction point, genistein mainly interacted with amino acid residues of LYS263, HIS266, ILE281, PHE264, ILE341, and ARG288 in the active site. And trans-resveratrol mainly interacted with amino acid residues of ARG288, ILE281, GLU343, and PHE287. Analysis of the mode of actions indicates that carbon hydrogen bond and π-alkyl stacked interactions made the higher agonistic activity of genistein than trans-resveratrol to PPARγ (Figure 4).

## 3. Discussion

The pathological mechanism of PH is very complicated. The main mechanism of action of single-target drugs currently used in clinical practice is the relaxation of pulmonary arteries. NO bioavailability is associated with the development of many different vascular diseases including PH. PDE-5 inhibitors, such as sildenafil, facilitate the vasodilating effects of endogenous NO. Riociguat acts as a soluble guanylate cyclase (sGC) stimulator by increasing cGMP biosynthesis through the direct stimulation of sGC in an NO-independent manner. In addition to the NO-related pathways, VEGF, eNOS, and COX-2 also play an important role in the pathogenesis of PH [19,20,21], which is consistent with our analysis. It is worth noting that drugs derived from natural products often have multi-target properties, which is precisely a characteristic of therapeutic drugs of complex diseases. 

The development of genomics, proteomics, metabonomics, interactomics (a recently developed field), and diseasomic has brought profound changes to modern medicine and drug research. However, with the extensive application of “OMICs” approaches, scientists are faced with increasing mass data, and hence, biologists and pharmacists require mathematic and computer skills for data analysis. This has led to the emergence of network pharmacology, an interdisciplinary field which combines traditional pharmacology, structural biology, computational science, and a series of OMICs approaches. To study the mechanism of PH, we could adopt the high-throughput gene chip method using samples collected from pulmonary artery smooth muscle cells, pulmonary artery endothelial cells, and pulmonary vascular tissues. We could compare the changes in the expression of differentially expressed genes in different animal models to determine the genes related to PH. Moreover, we can also use network pharmacology technology to explore data mining and network analysis. For complex diseases such as PH, network pharmacology has unique advantages; we employed this method to explore the anti-PH effect of genistein.

Firstly, the targets of genistein were determined according to different principles such as structural similarity and molecular docking, and genes related to PH were obtained from multiple databases. The 22 intersection genes of genistein and PH may be the potential targets of genistein against PH. The VarElect database was employed to analyze the correlation between potential targets and the “pulmonary hypertension” phenotypes. NOS2 and NOS3, the highest scored genes among the potential targets, were both related to the production of endogenous NO. Studies have shown that the expression of eNOS is significantly down-regulated in MCT-treated PH rats, which suggests that the NO signaling pathway could be suppressed after MCT injection [22,23,24]. Genistein could induce the activation of eNOS in a dose- and time-dependent manner [25]. In addition, genistein can ameliorate eNOS uncoupling in oxidized low-density lipoprotein-injured HUVECs [26]. We observed that PTGS2 was also closely related to the potency of genistein against PH. PTGS is the key enzyme in prostaglandin biosynthesis. PTGS1 (otherwise known as COX-1) is constitutively expressed in most cells, whereas PTGS2 (COX-2) is expressed in the condition of oxidative stress or during cytokine stimulation. Prostaglandin exerts a variety of pharmacological effects including vasodilation and inhibition of cell proliferation, which are closely related to the progression of PH [27,28]. Currently, prostacyclin analogues are still the mainstream drugs used for clinical treatment of PH [29]. The results also showed that PPARG is closely related to the effect of genistein against PH. PPARγ, which is coded by the PPARG gene, a nuclear receptor that functions as a transcription factor to regulate the pathology of numerous diseases [30]. The activation of PPARγ can suppress the proliferation of smooth muscle cells, reduce production of endothelin 1, and protect endothelial cells from apoptosis [31,32]. More importantly, it can promote the release of NO from endothelial cells by upregulating the activation of eNOS [33]. Interestingly, PGI2 and some of its analogs (such as iloprost and carbacyclin) can bind directly to PPARγ and lead to vasodilation, which partly explains the vasodilating effect of prostacyclin, as well as its cytoprotective properties [34]. According to the aforementioned results, PPARγ may be a key target of the therapeutic action of genistein.

GO and pathway analysis was also conducted using Metascape, and networks were constructed using Cytoscape 3.7.1. When exploring the mechanism of action of genistein, we focused on analyzing the biological processes and signaling pathways associated with cardiovascular diseases, especially PH. From the reactome pathways analysis, the highly enriched pathways of genistein were associated with NO synthesis (“eNOS activation”, “Metabolism of nitric oxide” and “eNOS activation and regulation”), inflammation (“Signaling by Interleukins”, “Toll Like Receptor 3 (TLR3) Cascade” and “MyD88:MAL(TIRAP) cascade initiated on plasma membrane”), VEGF pathway (VEGFA-VEGFR2 Pathway), and PTEN-PI3K/Akt pathway (“PIP3 activates AKT signaling”, and “PTEN Regulation”). These signaling pathways are critical in the progression of PH. Consistent results were also obtained by network topology analysis. Many genes related to NO synthesis (AKT1, NOS2, NOS3, and PPARG) also had higher topological parameter rankings. Inflammation is now increasingly recognized as a very important factor in the development of PH, especially in animal models of pre-clinical studies [35,36]. A recent study found that the innate immune receptor, TLR3, may act as a novel regulator of endothelial apoptosis in PH. TLR3 deficiency causes endothelial apoptosis and aggravates severe PH in mice [37]. Phosphatase-and-tensin homolog on chromosome 10 (PTEN) is a multifunctional lipid phosphatase that was initially identified as a tumor suppressor gene. Active PTEN protein serves as a modulator of several biological processes, including cell proliferation, apoptosis, and migration [38]. PTEN-PI3K/Akt pathway is obviously dysregulated in MCT- and hypoxia-treated animal models, and the up-regulation of the expression of PTEN could inhibit vascular remodeling [39]. From the analysis of the biological process, many genes were highly enriched in processes related to cell proliferation and apoptosis (“apoptotic signaling pathway”, “positive regulation of apoptotic process”, “cellular response to growth factor stimulus”, and “muscle cell proliferation”). One of the most obvious pathological changes in PH is a thickening in the tunica media of the pulmonary arterioles, mainly involving the proliferation and migration of pulmonary artery smooth muscle cells [40,41]. Consistent with the results of enrichment analysis, a large number of studies have shown that genistein could significantly inhibit hypoxia and growth factor-induced proliferation of pulmonary artery smooth muscle cells [10,42,43]. From the molecular function analysis, many genes were significantly enriched in the “nitric-oxide synthase regulator activity”. The results of GO and pathway analysis were also consistent with the correlation between targets and phenotype in VarElect analysis.

We used molecular docking to explore the mechanism of genistein. In addition to the results of VarElect analysis, we found that PPARγ had a high correlation with “pulmonary hypertension” phenotype in potential targets of genistein against PH. Furthermore, the results of network analysis show that PPARγ was widely involved in “muscle cell proliferation”, “PIP3 activates AKT signaling”, and “PTEN Regulation” and other processes in the main signaling pathways associated with the action of genistein against PH. Finally, PPARγ is of interest as a target in the treatment of PH [17,20], and genistein is considered a nature-derived PPAR agonist [44,45]. To investigate the mechanism of the interactions between genistein and PPARγ, molecular docking method was employed to validate the reliability of the interaction. The result indicates that genistein can bind with the large binding site of PPARγ with good binding scores in respect to trans-resveratrol. The exact mechanism based on molecular docking also requires further validation in biological experiments.

Our study had several limitations. Firstly, the data set of the PH-related genes we used was based on existing research findings. Although it is beneficial to explore the effect of genistein on the basis of existing findings, it hinders the discovery of new targets in the treatment of PH, which is not fully studied at present. Secondly, based on the topological analysis and key targets analysis of the constructed network, we only docked PPARγ with genistein. In the follow-up study, we should not only use biological methods to verify the binding ability of genistein with PPARγ, but also analyze the mechanism with other key targets based on the results of further biological experiments.

Overall, this study demonstrated that genistein may exert an anti-PH effect through multiple signaling pathways. Although further studies are necessary to elucidate the precise mechanism, this current study provides a systematic and visual overview of possible targets and signaling pathways associated with the activities of genistein against PH.

## 4. Materials and Methods

**Figure 5:**
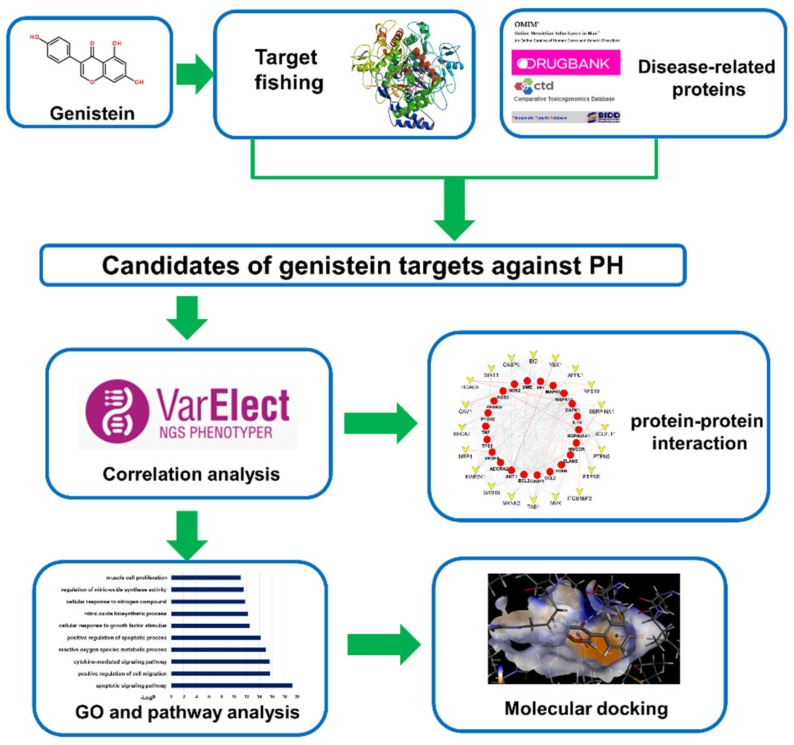
Work scheme of network pharmacology approach. PH: pulmonary hypertension; GO: Gene ontology.

The work scheme is depicted in Figure 5. Briefly speaking, through database mining and a range of online tools, two types of targets were identified: (1) targets of genistein, and (2) genes related to PH. The intersection of these two groups of targets may be part of the potential anti-PH targets of genistein. Then the phenotype correlation analysis of PH, protein-protein interaction (PPI) analysis, and Gene Ontology (GO) analysis were performed on the obtained targets. Beyond that, the target-pathway/function network was constructed to obtain the key targets and pathways of genistein. Lastly, molecular docking was performed to predict the mechanism of interaction with PPARγ.

### 4.1. Druglikeness Prediction

Based on Lipinski’s rule of five (RO5), a series of chemical properties and physical properties of genistein were investigated according to the PubChem database (pubchem.ncbi.nlm.nih.gov). The parameters include molecular weight (MW), XLogP3 (octanol-water partition coefficient), topological polar surface area (TPSA), number of rotatable bonds, hydrogen bond acceptor count, and hydrogen bond donor count. To further observe the ADMET properties of genistein, the ADMET descriptor in Discovery Studio 2016 was employed to analyze the absorption properties as previously described [46], such as human intestinal absorption (HIA) and blood brain barrier (BBB) plots.

### 4.2. Predicting Targets of Genistein

A variety of approaches based on information integration, principle of structural similarity, and reverse docking technology were introduced to discover the targets of genistein. More concretely, the potential targets were collected from STITCH database [47], Traditional Chinese Medicine Systems Pharmacology Database (TCMSP) [48], Bindingdatabase (BindingDB) [49,50], Swiss Target Prediction [51], and PharmMapper Server [52]. In STITCH and the TCMSP database, the compound’s name (genistein) was used for target searching. According to the principle of structural similarity, we obtained predicted targets by SMILES string of genistein (C1=CC(=CC=C1C2=COC3=CC(=CC(=C3C2=O)O)O)O) in BindingDB and the Swiss Target Prediction database. In addition, the SDF structure file of genistein (PubChem CID: 5280961) was imported into PharmMapper for potential target using the pharmacophore mapping approach. Afterward, the obtained targets were also sent to the UniProt Database (http://www.uniprot.org/) for normalization. Finally, the targets from different databases were merged and the duplicated targets were removed (Table S1). In the following description, we call this part of the gene set “targets of genistein”.

### 4.3. Collecting Targets Related to Pulmonary Hypertension

To ensure the comprehensiveness of disease-related genes collected, PH-associated targets were extracted from the Online Mendelian Inheritance in Man (OMIM) database (https://www.omim.org/), DrugBank database (http://www.drugbank.ca/), MalaCards database (https://www.malacards.org/), Therapeutic Target Database (TTD, http://bidd.nus.edu.sg/group/cjttd/), Comparative Toxicogenomics Database (http://ctdbase.org/) and CooLGeN database (http://ci.smu.edu.cn/CooLGeN/). In the above databases, set the keyword of the phenotype to “pulmonary hypertension”. In the CooLGeN database, targets with hit scores greater than 5 were taken. In the OMIM and MalaCards database, due to the diversity of PH classification, genes involved in different PH disease types (PPH1, PPH2, PPH3 and PPH4) were integrated. Finally, 243 PH-related targets were discovered from the above databases after deleting redundancy. In the following description, we call this part of the gene set “genes related to PH”. The detailed information about these targets was described in Table S2. The intersection of these two groups of targets may be part of the potential anti-PH targets of genistein. In the following, we call this part of the intersection genes “intersection genes of genistein and PH”.

### 4.4. Phenotypic Association Analysis of Genistein Targets and Pulmonary Hypertension

The VarElect database allows for rapid screening of genes that are related to a certain phenotype/disease directly or indirectly [53]. A central function of VarElect is its capacity to perform gene list interpretation and scoring based on user-entered phenotype keywords. The scores included in VarElect results basically stem from Elasticsearch technology. The score of a specific term is determined by the frequency of its appearance in the individual Genecards, as compared to that in all genes (inverse document frequency correction). We used the VarElect online tool to analyze the correlation between “the intersection genes of genistein and PH” and the phenotype of “pulmonary hypertension”.

### 4.5. Protein-Protein Interaction Analysis

The GeneMANIA tool can not only construct the PPI network, but also find a series of genes related to the inputted genes based on a large number of functional related data, and analyze the interaction between them, such as co-expression and co-localization [54]. In the current study, GeneMANIA database was employed to construct a protein-protein interaction network of intersection genes of genistein and PH, and to explore the genes associated with them. Through GeneMANIA analysis, we can get not only the relationship between the inputted intersection genes, but also other targets which are closely related to these genes. In the following analysis, we call this new set of genes “predicted targets of genistein against PH”. Topological properties of the PPI network, including average shortest path length, betweenness centrality, degree and closeness centrality were determined using the Network Analyzer tool in Cytoscape 3.7.1

### 4.6. Gene Ontology Enrichment Analysis

Metascape is a comprehensive tool for gene annotation and enrichment analysis. It integrates multiple databases such as GO, KEGG, UniProt, and DrugBank [55]. Metascape was used for GO analysis and Reactome pathway analysis of the predicted targets against PH. The predicted targets of genistein against PH were mapped and classified into different types of GO category or pathways. The results closely related to the mechanism of PH were included for further analysis in light of the pathological and nearness analysis data. The pathways and biological processes related to the mechanism of PH can be obtained from the approaches as follows: Firstly, the targets of genistein against PH were collected to be inputted into the Kyoto Encyclopedia of Genes and Genomes database (KEGG, http://www.kegg.jp/) and the Database for Annotation, Visualization, and Integrated Discovery (DAVID) v6.8 tool (https://david.ncifcrf.gov/home.jsp) to acquire the information of signaling pathways. Secondly, the subsequent pathways related to PH and pulmonary vascular remodeling were picked out based on the pathological and clinical data.

### 4.7. Target-Pathway/Function Network Construction

In this section, the network was constructed by Cytoscape 3.7.1, an open source software platform for visualizing complex networks. In the network, potential targets of genistein for treating PH, biological processes, and signaling pathways obtained through enrichment analysis were represented by nodes, and the interactions between them were represented by edges. 

### 4.8. Molecular Docking

To gain an insight into the relationship between genistein and a key target in the network, molecular docking was performed to assess the strength and mode of interactions between genistein and PPARγ. The protein crystal structure of PPARγ was obtained from Protein Data Bank (PDB ID: 4JAZ). 4JAZ represents the complex of PPARγ with trans-resveratrol. The conformation of trans-resveratrol in the crystal structure was used as a reference for docking analysis of genistein. The process is simply described as follows: Discovery Studio 2016 was applied for molecular docking. After the protein was cleaned to remove the effect of water molecules and add missing atoms to repair the N-and C-terminal residues, trans-resveratrol was selected to build the binding site. Then we ran CDOCKER process to genistein and trans-resveratrol. The interaction energy and the mode of action were analyzed after that.

## Figures and Tables

**Table 1:** Molecular properties of genistein.

Property	Parameter
MW	270.24 g/mol
PSA	87 ^A
XLogP3-AA	2.7
H-bond donor	3
H-bond acceptor	5
Rotatable bond count	1
ADMET Absorption Level	0
BBB Level	3

MW: molecular weight; PSA: polar surface area; XLogP3-AA: computed octanol/water partition coefficient; BBB Level: blood brain barrier level.

**Table 2:** VarElect analysis of targets of genistein with the “pulmonary hypertension” phenotype.

NO.	Symbol	Description	Phenotypic Correlation	Score
1	NOS3	Nitric Oxide Synthase 3	Directly	20.12
2	NOS2	Nitric Oxide Synthase 2	Directly	16.1
3	VEGFA	Vascular Endothelial Growth Factor A	Directly	11.77
4	TNF	Tumor Necrosis Factor	Directly	10.34
5	PTGS2	Prostaglandin-Endoperoxide Synthase 2	Directly	8.69
6	ELANE	Elastase, Neutrophil Expressed	Directly	8.53
7	CCL2	C-C Motif Chemokine Ligand 2	Directly	7.54
8	PPARG	Peroxisome Proliferator Activated Receptor Gamma	Directly	7.45
9	IL1B	Interleukin 1 Beta	Directly	6.44
10	EGFR	Epidermal Growth Factor Receptor	Directly	3.69
11	MIF	Macrophage Migration Inhibitory Factor	Directly	3.57
12	TP53	Tumor Protein P53	Directly	3.16
13	AKT1	AKT Serine/Threonine Kinase 1	Directly	2.53
14	CASP3	Caspase 3	Directly	2.38
15	MAPK1	Mitogen-Activated Protein Kinase 1	Directly	1.98
16	HSP90AA1	Heat Shock Protein 90 Alpha Family Class A Member 1	Directly	1.91
17	HMGCR	3-Hydroxy-3-Methylglutaryl-CoA Reductase	Directly	1.83
18	MME	Membrane Metalloendopeptidase	Directly	0.64
19	MAPK3	Mitogen-Activated Protein Kinase 3	Directly	0.58
20	BCL2	BCL2, Apoptosis Regulator	Directly	0.29
21	MAPK14	Mitogen-Activated Protein Kinase 14	InDirectly	8.24
22	ADORA2A	Adenosine A2a Receptor	InDirectly	3.27

The intersection genes are divided into two parts that are directly related and indirectly related to “pulmonary hypertension”. The level of the score indicates the correlation between genes and the target phenotype.

**Table 3:** The CDOCKER analysis results of genistein and trans-resveratrol at the active site of PPARγ.

Compound	-CDCOKER Energy (kcal/mol)	-CDCOKER Interaction_Energy (kcal/mol)
Genistein	29.4126	37.9578
Trans-resveratrol	25.1709	33.4104

## Supplementary Materials

Supplementary materials can be found at https://www.mdpi.com/1422-0067/20/22/5569/s1.
