# Supplementary material for: Systematic Elucidation of the Mechanism of Genistein against Pulmonary Hypertension via Network Pharmacology Approach
Source: Int J Mol Sci. 2019 Nov 7;20(22):5569. doi: 10.3390/ijms20225569 (PMC6888439; doi:10.3390/ijms20225569)
Supplement: Supplementary file 1 [file ijms-20-05569-s001.pdf]

Table S1. Targets of genistein

Gene symbol	UniProt ID	Source
ABCB1	MDR1_HUMAN	BindingDB
ABCG2	ABCG2_HUMAN	SwissTargetPrediction
ADAM33	ADA33_HUMAN	PharmMapper
ADORA1	AA1R_HUMAN	SwissTargetPrediction
ADORA2A	AA2AR_HUMAN	SwissTargetPrediction
AKR1B1	ALDR_HUMAN	PharmMapper/TCMSP
AKR1C2	AK1C2_HUMAN	PharmMapper
AKT1	AKT1_HUMAN	STITCH
ALDH2	ALDH2_HUMAN	BindingDB
AOX1	AOXA_HUMAN	BindingDB
AR	ANDR_HUMAN	STITCH/PharmMapper/TCMSP
BACE1	BACE1_HUMAN	PharmMapper
BCL2	BCL2_HUMAN	TCMSP
BTK	BTK_HUMAN	TCMSP
CA12	CAH12_HUMAN	BindingDB
CA2	CAH2_HUMAN	PharmMapper
CA4	CAH4_HUMAN	BindingDB
CA7	CAH7_HUMAN	BindingDB
CASP3	CASP3_HUMAN	PharmMapper
CCL2	CCL2_HUMAN	TCMSP
CCNA2	CCNA2_HUMAN	PharmMapper
CDK1	CDK1_HUMAN	TCMSP
CDK2	CDK2_HUMAN	TCMSP/PharmMapper
CFTR	CFTR_HUMAN	STITCH/TCMSP
CHEK1	CHK1_HUMAN	TCMSP
CRABP2	RABP2_HUMAN	PharmMapper
CTSK	CATK_HUMAN	PharmMapper
CYP19A1	CP19A_HUMAN	STITCH/BindingDB
CYP1A1	CP1A1_HUMAN	STITCH
DCK	DCK_HUMAN	PharmMapper
DHODH	PYRD_HUMAN	PharmMapper
DRD5	DRD5_HUMAN	BindingDB
EGFR	EGFR_HUMAN	SwissTargetPrediction/BindingDB/TCMSP
ELANE	ELNE_HUMAN	PharmMapper
ER	Q99893_HUMAN	TCMSP
ERBB2	ERBB2_HUMAN	SwissTargetPrediction
ERBB3	ERBB3_HUMAN	SwissTargetPrediction
ERBB4	ERBB4_HUMAN	SwissTargetPrediction
ESR1	ESR1_HUMAN	PharmMapper/STITCH/Swiss

		TargetPrediction
ESR2	ESR2_HUMAN	PharmMapper/STITCHSwissTargetPrediction/BindingDB
ESRRA	ERR1_HUMAN	SwissTargetPrediction/BindingDB
ESRRB	ERR2_HUMAN	SwissTargetPrediction
ESRRG	ERR3_HUMAN	PharmMapper
FGFR1	FGFR1_HUMAN	PharmMapper
FGG	FIBG_HUMAN	PharmMapper
FOXO3	FOXO3_HUMAN	STITCH
GCK	HXK4_HUMAN	PharmMapper/TCMSP
GLB1	BGAL_HUMAN	TCMSP
GSTA1	GSTA1_HUMAN	PharmMapper
GSTM2	GSTM2_HUMAN	PharmMapper
GSTP1	GSTP1_HUMAN	PharmMapper
HDAC6	HDAC6_HUMAN	TCMSP
HMGCR	HMDH_HUMAN	PharmMapper/TCMSP
HPGD	PGDH_HUMAN	TCMSP
HRAS	RASH_HUMAN	PharmMapper
HSD11B1	DHI1_HUMAN	PharmMapper
HSD17B1	DHB1_HUMAN	PharmMapper/SwissTargetPrediction
HSP90AA1	HS90A_HUMAN	PharmMapper/TCMSP
IL1B	IL1B_HUMAN	TCMSP
INS	INS_HUMAN	TCMSP
JUN	JUN_HUMAN	TCMSP
KCNJ11	KCJ11_HUMAN	TCMSP
LDLR	LDLR_HUMAN	TCMSP
MAPK1	MK01_HUMAN	TCMSP
MAPK14	MK14_HUMAN	PharmMapper/TCMSP
MAPK3	MK03_HUMAN	TCMSP
MAPT	TAU_HUMAN	SwissTargetPrediction
MET	MET_HUMAN	PharmMapper
METAP2	MAP2_HUMAN	PharmMapper
MIF	MIF_HUMAN	BindingDB
MME	NEP_HUMAN	PharmMapper
MMP3	MMP3_HUMAN	PharmMapper
NOS2	NOS2_HUMAN	PharmMapper/TCMSP
NOS3	NOS3_HUMAN	STITCH
NR1H4	NR1H4_HUMAN	PharmMapper
OAT	OAT_HUMAN	PharmMapper
PCK1	PCKGC_HUMAN	PharmMapper
PDE4D	PDE4D_HUMAN	PharmMapper
PIK3CG	PK3CG_HUMAN	TCMSP

PLK1	PLK1_HUMAN	TCMSP
PPARG	PPARG_HUMAN	STITCH/TCMSP
PRKACA	KAPCA_HUMAN	PharmMapper/TCMSP
PRSS1	TRY1_HUMAN	TCMSP
PTGFR	PF2R_HUMAN	TCMSP
PTGS1	PGH1_HUMAN	TCMSP
PTGS2	PGH2_HUMAN	TCMSP
PTPN1	PTN1_HUMAN	PharmMapper
RARG	RARG_HUMAN	PharmMapper
RDH8	RDH8_HUMAN	SwissTargetPrediction
SELE	LYAM2_HUMAN	TCMSP
SHBG	SHBG_HUMAN	PharmMapper
SHMT1	GLYC_HUMAN	PharmMapper
SULT1E1	ST1E1_HUMAN	TCMSP
SULT2B1	ST2B1_HUMAN	PharmMapper
SYK	KSYK_HUMAN	TCMSP
TDP1	TYDP1_HUMAN	SwissTargetPrediction
TNF	TNFA_HUMAN	TCMSP
TP53	P53_HUMAN	TCMSP
TTR	TTHY_HUMAN	PharmMapper
UCK2	UCK2_HUMAN	PharmMapper
VCAM1	VCAM1_HUMAN	TCMSP
VDR	VDR_HUMAN	PharmMapper
VEGFA	VEGFA_HUMAN	TCMSP
WARS	SYWC_HUMAN	PharmMapper

Table S2. Pulmonary hypertension related targets.

Gene symbol	UniProt ID	Source
ABCA3	ABCA3_HUMAN	DrugBank/CTD
ACE	ACE_HUMAN	CooLGeN/CTD
ACE2	ACE2_HUMAN	CooLGeN
ACO1	ACOC_HUMAN	CooLGeN
ACTA2	ACTA_HUMAN	CooLGeN
ACVRL1	ACVL1_HUMAN	MalaCards/CooLGeN/CTD
ADIPOQ	ADIPO_HUMAN	CooLGeN
ADM	ADML_HUMAN	CooLGeN
ADORA2A	AA2AR_HUMAN	CooLGeN
AGER	RAGE_HUMAN	CooLGeN
AGT	ANGT_HUMAN	CooLGeN
AGTR1	AGTR1_HUMAN	CooLGeN
AKT1	AKT1_HUMAN	CooLGeN
ALB	ALBU_HUMAN	CooLGeN
ALOX5	LOX5_HUMAN	CTD

ANGPT1	ANGP1_HUMAN	CooLGeN
APLN	APEL_HUMAN	CooLGeN
APLNR	APJ_HUMAN	CooLGeN
APOE	APOE_HUMAN	CooLGeN
ARG2	ARGI2_HUMAN	CTD
ASIC1	ASIC1_HUMAN	CooLGeN
ATP7A	ATP7A_HUMAN	CTD
AVP	NEU2_HUMAN	CooLGeN
BCL2	BCL2_HUMAN	CooLGeN
BIRC5	BIRC5_HUMAN	CooLGeN
BMP2	BMP2_HUMAN	CooLGeN
BMP4	BMP4_HUMAN	CooLGeN
BMPR1A	BMR1A_HUMAN	CooLGeN
BMPR2	BMPR2_HUMAN	OMIM/CooLGeN/MalaCards/CTD
BRD4	BRD4_HUMAN	CTD
CA9	CAH9_HUMAN	CTD
CALCA	CALCA_HUMAN	CTD/CooLGeN
CARD8	CARD8_HUMAN	CooLGeN
CASP3	CASP3_HUMAN	CooLGeN
CASR	CASR_HUMAN	CooLGeN
CAT	CATA_HUMAN	CooLGeN
CAV1	CAV1_HUMAN	MalaCards/OMIM/CooLGeN/CTD
CBLN2	CBLN2_HUMAN	CTD
CCL1	CCL1_HUMAN	CTD
CCL2	CCL2_HUMAN	CTD/CooLGeN
CCL21	CCL21_HUMAN	CooLGeN
CD40LG	CD40L_HUMAN	CTD
CDKN1A	CDN1A_HUMAN	CooLGeN
CMA1	CMA1_HUMAN	CooLGeN
COX8A	COX8A_HUMAN	CooLGeN
CP	CERU_HUMAN	DrugBank
CPS1	CPSM_HUMAN	CTD
CRP	CRP_HUMAN	CooLGeN
CTGF	CTGF_HUMAN	CooLGeN
CX3CL1	X3CL1_HUMAN	CooLGeN
CXCL12	SDF1_HUMAN	CooLGeN
CXCL8	IL8_HUMAN	CTD/CooLGeN
CXCR4	CXCR4_HUMAN	CooLGeN
CYSLTR2	CLTR2_HUMAN	DrugBank
DDAH1	DDAH1_HUMAN	CooLGeN
DNM1L	DNM1L_HUMAN	CooLGeN
DRD2	DRD2_HUMAN	TTD

ECE1	ECE1_HUMAN	CooLGeN
EDN1	EDN1_HUMAN	MalaCards/CooLGeN/CTD
EDN3	EDN3_HUMAN	CooLGeN
EDNRA	EDNRA_HUMAN	TTD/CooLGeN
EDNRB	EDNRB_HUMAN	TTD/CooLGeN/CTD
EGFR	EGFR_HUMAN	CooLGeN
EGLN1	EGLN1_HUMAN	CooLGeN
EGR1	EGR1_HUMAN	CooLGeN
EGR3	EGR3_HUMAN	CooLGeN
EIF2AK4	E2AK4_HUMAN	
ELANE	ELNE_HUMAN	CooLGeN
ELN	ELN_HUMAN	CooLGeN
ENG	EGLN_HUMAN	MalaCards/CooLGeN
ENTPD1	ENTP1_HUMAN	CooLGeN
EPAS1	EPAS1_HUMAN	CooLGeN
EPHX2	HYES_HUMAN	CooLGeN
EPO	EPO_HUMAN	CooLGeN
F3	TF_HUMAN	CooLGeN
F5	FA5_HUMAN	CooLGeN
FBLN5	FBLN5_HUMAN	CTD
FGF2	FGF2_HUMAN	CooLGeN
FLT1	VGFR1_HUMAN	CooLGeN
FOXF1	FOXF1_HUMAN	MalaCards
FOXO1	FOXO1_HUMAN	CTD/CooLGeN
G6PD	G6PD_HUMAN	CooLGeN
GBA	GLCM_HUMAN	CTD
GDF15	GDF15_HUMAN	CooLGeN
GDF2	GDF2_HUMAN	CTD/CooLGeN
GHRL	GHRL_HUMAN	CooLGeN
GREM1	GREM1_HUMAN	CooLGeN
GRP	GRP_HUMAN	CooLGeN
GUCY1A1	GCYA1_HUMAN	CTD
GUCY2D	GUC2D_HUMAN	TTD
HBG2	HBG2_HUMAN	CooLGeN
HDAC1	HDAC1_HUMAN	CTD
HDAC4	HDAC4_HUMAN	CTD
HDAC5	HDAC5_HUMAN	CTD
HGF	HGF_HUMAN	CooLGeN
HIF1A	HIF1A_HUMAN	TTD/CTD/CooLGeN
HMGB1	HMGB1_HUMAN	CooLGeN
HMGCR	HMDH_HUMAN	CooLGeN
HMOX1	HMOX1_HUMAN	CooLGeN
HSP90AA1	HS90A_HUMAN	CooLGeN
HSPA4	HSP74_HUMAN	CooLGeN

HTR1A	5HT1A_HUMAN	DrugBank
HTR1B	5HT1B_HUMAN	DrugBank
HTR1D	5HT1D_HUMAN	DrugBank
HTR1E	5HT1E_HUMAN	DrugBank
HTR1F	5HT1F_HUMAN	DrugBank
HTR2A	5HT2A_HUMAN	DrugBank
HTR2B	5HT2B_HUMAN	DrugBank/TTD/CooLGeN/CTD
HTR2C	5HT2C_HUMAN	DrugBank
HTR3A	5HT3A_HUMAN	DrugBank
HTR3B	5HT3B_HUMAN	DrugBank
HTR3C	5HT3C_HUMAN	DrugBank
HTR3D	5HT3D_HUMAN	DrugBank
HTR3E	5HT3E_HUMAN	DrugBank
HTR4	5HT4R_HUMAN	DrugBank
HTR6	5HT6R_HUMAN	DrugBank
HTR7	5HT7R_HUMAN	DrugBank
ICAM1	ICAM1_HUMAN	CooLGeN
IFNA1	IFNA1_HUMAN	CooLGeN
IFNB1	IFNB_HUMAN	CooLGeN
IGF1	IGF1_HUMAN	CooLGeN
IL10	IL10_HUMAN	CooLGeN
IL13	IL13_HUMAN	CooLGeN
IL1B	IL1B_HUMAN	CooLGeN
IL6	IL6_HUMAN	CooLGeN
KANTR	A0A1W2PQU2_HUMAN	CooLGeN
KCNA3	KCNA3_HUMAN	CooLGeN
KCNA5	KCNA5_HUMAN	CTD/CooLGeN
KCNB1	KCNB1_HUMAN	DrugBank/CooLGeN
KCNK3	KCNK3_HUMAN	OMIM/CooLGeN/MalaCards/CTD
KCNMA1	KCMA1_HUMAN	CTD
KDR	VGFR2_HUMAN	CooLGeN
KIT	KIT_HUMAN	CooLGeN
KNG1	KNG1_HUMAN	CooLGeN
LCN2	NGAL_HUMAN	CooLGeN
LEP	LEP_HUMAN	CooLGeN
LGALS3	LEG3_HUMAN	CooLGeN
LOX	LYOX_HUMAN	CTD
LPA	APOA_HUMAN	CooLGeN
LRP1	LRP1_HUMAN	CooLGeN
MADH9	SMAD9_HUMAN	OMIM
MAPK1	MK01_HUMAN	CooLGeN
MAPK14	MK14_HUMAN	CooLGeN

MAPK3	MK03_HUMAN	CooLGeN
MIF	MIF_HUMAN	CooLGeN
MME	NEP_HUMAN	CooLGeN
MMP1	MMP1_HUMAN	CooLGeN
MMP2	MMP2_HUMAN	CooLGeN
MMP9	MMP9_HUMAN	CooLGeN
MPO	PERM_HUMAN	CooLGeN
MPV17	MPV17_HUMAN	CooLGeN
MTOR	MTOR_HUMAN	CooLGeN
NF1	NF1_HUMAN	CooLGeN
NFATC2	NFAC2_HUMAN	CTD
NFATC3	NFAC3_HUMAN	CooLGeN
NFKB1	NFKB1_HUMAN	CooLGeN
NOS1	NOS1_HUMAN	CooLGeN
NOS2	NOS2_HUMAN	CooLGeN
NOS3	NOS3_HUMAN	TTD/CTD/CooLGeN
NOTCH1	NOTC1_HUMAN	CooLGeN
NOTCH3	NOTC3_HUMAN	CooLGeN
NOX4	NOX4_HUMAN	CooLGeN
NPPA	ANF_HUMAN	CooLGeN
NPPB	ANFB_HUMAN	MalaCards/CooLGeN/CTD
NPPC	ANFC_HUMAN	CooLGeN
NPR1	ANPRA_HUMAN	CooLGeN
PDE5A	PDE5A_HUMAN	CooLGeN
PDGFRB	PGFRB_HUMAN	CooLGeN
PECAM1	PECA1_HUMAN	CooLGeN
PF4	PLF4_HUMAN	CooLGeN
PGF	PLGF_HUMAN	CooLGeN
PLAT	TPA_HUMAN	CooLGeN
PPARG	PPARG_HUMAN	CooLGeN
PPIA	PPIA_HUMAN	CooLGeN
PRKG1	KGP1_HUMAN	CTD
PROC	PROC_HUMAN	CooLGeN
PTEN	PTEN_HUMAN	CooLGeN
PTGER2	PE2R2_HUMAN	TTD
PTGIR	PI2R_HUMAN	TTD
PTGIS	PTGIS_HUMAN	CooLGeN
PTGS2	PGH2_HUMAN	CooLGeN
PTX3	PTX3_HUMAN	CooLGeN
REN	RENI_HUMAN	CooLGeN
RETN	RETN_HUMAN	CooLGeN
RETNLB	RETNB_HUMAN	CooLGeN
RHOA	RHOA_HUMAN	CooLGeN
ROCK1	ROCK1_HUMAN	TTD

ROCK2	ROCK2_HUMAN	CooLGeN
RPL5	RL5_HUMAN	MalaCards
S100A4	S10A4_HUMAN	CooLGeN
S1PR1	S1PR1_HUMAN	DrugBank
SERPINA1	A1AT_HUMAN	CooLGeN
SERPINE1	PAI1_HUMAN	CooLGeN
SFTPA1	SFTA1_HUMAN	DrugBank
SFTPD	SFTPD_HUMAN	DrugBank
SGK1	SGK1_HUMAN	DrugBank
SLC31A1	COPT1_HUMAN	CTD
SLC6A4	SC6A4_HUMAN	CTD/CooLGeN
SLC8A1	NAC1_HUMAN	CooLGeN
SMAD1	SMAD1_HUMAN	CooLGeN
SMAD2	SMAD2_HUMAN	CooLGeN
SMAD3	SMAD3_HUMAN	CooLGeN
SMAD9	SMAD9_HUMAN	MalaCards/CooLGeN/CTD
SOD1	SODC_HUMAN	CooLGeN
SOD2	SODM_HUMAN	CTD/CooLGeN
SOD3	SODE_HUMAN	CooLGeN
SPHK1	SPHK1_HUMAN	CooLGeN
SPP1	OSTP_HUMAN	CooLGeN
SRC	SRC_HUMAN	CooLGeN
STAT3	STAT3_HUMAN	CooLGeN
TAC1	TKN1_HUMAN	CooLGeN
TBX4	TBX4_HUMAN	CooLGeN
TEK	TIE2_HUMAN	CooLGeN
TGFA	TGFA_HUMAN	CTD/CooLGeN
TGFB1	TGFB1_HUMAN	CooLGeN
TGM2	TGM2_HUMAN	CooLGeN
THBD	TRBM_HUMAN	CooLGeN
THBS1	TSP1_HUMAN	CooLGeN
TIMP1	TIMP1_HUMAN	CooLGeN
TLR4	TLR4_HUMAN	CooLGeN
TNC	TENA_HUMAN	CooLGeN
TNF	TNFA_HUMAN	CTD/CooLGeN
TNFRSF11B	TR11B_HUMAN	CooLGeN
TNNI3	TNNI3_HUMAN	CooLGeN
TNXA	TENXA_HUMAN	MalaCards
TP53	P53_HUMAN	CooLGeN
TPH1	TPH1_HUMAN	CTD/CooLGeN
TRPC1	TRPC1_HUMAN	CooLGeN
TRPC6	TRPC6_HUMAN	CooLGeN
TRPV4	TRPV4_HUMAN	CooLGeN
UTS2	UTS2_HUMAN	CooLGeN

VEGFA	VEGFA_HUMAN	CooLGeN
VIP	VIP_HUMAN	CooLGeN
VIPR1	VIPR1_HUMAN	TTD
YAP1	YAP1_HUMAN	CooLGeN

Table S3. 42 nodes with average shortest path length, betweenness centrality and closeness centrality.

Gene name	Average Shortest Path Length	Betweenness Centrality	Closeness Centrality	Degree
MAPK1	1.463415	0.127107	0.683333	1
MAPK14	1.682927	0.045456	0.594203	29
MAPK3	1.634146	0.072224	0.61194	6
TP53	1.560976	0.072095	0.640625	20
AKT1	1.658537	0.055959	0.602941	10
HSP90AA1	1.560976	0.121682	0.640625	14
CAV1	1.731707	0.050278	0.577465	7
EGFR	1.756098	0.012622	0.569444	21
CASP3	1.853659	0.043363	0.539474	16
BCL2	1.878049	0.006759	0.532468	24
MAP2K1	1.829268	0.022899	0.546667	7
PTGS2	1.756098	0.04724	0.569444	21
TNF	1.756098	0.024234	0.569444	2
CASP9	1.902439	0.034011	0.525641	10
PPARG	1.853659	0.01413	0.539474	5
VEGFA	1.756098	0.032006	0.569444	7
NOS3	1.804878	0.017202	0.554054	28
BID	1.926829	0.003552	0.518987	14
IL1B	1.829268	0.031844	0.546667	1
MKNK2	1.97561	0.004953	0.506173	20
SIRT1	1.926829	0.011866	0.518987	50
TAB1	2.02439	0.001054	0.493976	39
PTPRR	2.146341	0.00185	0.465909	32
GRB10	2.02439	0.007942	0.493976	9
BCL2L11	1.95122	0.008546	0.5125	13
NOS2	2	0.035282	0.5	5
MIF	1.926829	0.017195	0.518987	7
PTPN5	2.097561	0.001166	0.476744	9
YBX1	1.95122	0.015612	0.5125	15
CCL2	2.219512	0.007366	0.450549	4
HMGCR	2.365854	0.006911	0.42268	15
MVK	2.121951	0.055317	0.471264	17
SERPINA1	2.219512	0.020488	0.450549	8

BRCA2	2.04878	0.015497	0.488095	10
APPL1	2.365854	0	0.42268	5
RPS19	2.439024	3.05E-04	0.41	7
MME	2.146341	0.005724	0.465909	11
HDAC4	2.219512	8.15E-04	0.450549	10
NRP1	2.414634	0.001631	0.414141	16
ELANE	2.780488	6.98E-04	0.359649	30
ADORA2A	3.097561	0	0.322835	15
ITGB1BP2	2.536585	0	0.394231	8
